# Exclusive breastfeeding among city-dwelling professional working mothers in Ghana

**DOI:** 10.1186/s13006-016-0083-8

**Published:** 2016-09-06

**Authors:** Elvis J. Dun-Dery, Amos K. Laar

**Affiliations:** Department of Population, Family and Reproductive Health, School of Public Health, College of Health Sciences, University of Ghana, Accra, Ghana

**Keywords:** Exclusive breastfeeding, Breastfeeding practice, Professional working mothers, Ghana

## Abstract

**Background:**

In Ghana, periodic national surveys report the practice of exclusive breastfeeding (EBF) in the general population to be over 50 %. However, little is known about EBF among professional working mothers, particularly its duration after maternity leave. Female workers are entitled to 12 weeks (84 days) of maternity leave with full pay in Ghana, and this can be extended by two additional weeks in case of a caesarean or abnormal delivery. This study assessed the prevalence of EBF, as well as factors associated with the practice among professional working mothers in one of the ten regional capitals of Ghana.

**Methods:**

The study was descriptive cross-sectional in design and employed a multi-stage sampling technique to sample 369 professional working mothers. The study was planned and implemented between January to July 2015. Study-specific structured questionnaires were used in the data collection over a period of one month. Some factors including demographic characteristics, types of facilities available at workplace to support breastfeeding, challenges to exclusive breastfeeding at the workplace and mother’s knowledge base on EBF, were assessed. Exclusive breastfeeding is defined as feeding infants with only breast milk, without supplemental liquids or solids except for liquid medicine and vitamin or mineral supplements.

**Results:**

There was a near universal awareness of exclusive breastfeeding among respondents (99 %). Even though most mothers initiated breastfeeding within an hour of delivery (91 %), the EBF rate at six months was low (10.3 %). The study identified three elements as determinants of EBF; Those who did not receive infant feeding recommendation from health workers were less likely to practice exclusive breastfeeding (Adjusted Odds Ratio [AOR] 0.45; 95 % Confidence Interval [CI] 0.27, 0.77), mothers who had shorter duration of maternity leave were less likely to practice exclusive breastfeeding (AOR 0.09; 95 % CI 0.02, 0.45), and those who had a normal delivery were almost 10 times as likely to practice exclusive breastfeeding (AOR 9.02; 95 % CI 2.85, 28.53).

**Conclusion:**

Given the high breastfeeding initiation, but low EBF continuation rate among professional working mothers, improved policies around maternity leave and breastfeeding friendly work environments are needed.

## Background

The 2008 Lancet Series on Maternal and Child Undernutrition indicated that suboptimum breastfeeding, especially not exclusively breastfeeding a child for the first six months of life, results in 1.4 million deaths and 10 % of the disease burden in children younger than five years in low-income and middle-income countries [1]. Other statistics indicate that one hundred and thirty-five million babies are delivered annually, but only 42 % (57 million) initiate breastfeeding within the first hour after birth, 39 % are breastfed exclusively during the first six months, and 58 % continue breastfeeding up to the age of two years [2]. Several studies have reported barriers accounting for this situation, including returning to work after delivery [3]. Others have stated factors that determine the success of exclusive breastfeeding even upon return to work, indicating that a supportive workplace and working environment are essential [4]. Yet, the Ghana 2010 Population and Housing Census Report showed an increasing trend of women joining the labour force [5]. Guendelman et al. note that the challenge of balancing breastfeeding and paid work is an important reason for breastfeeding cessation in the first six months [6]. In Ghana, the success of exclusive breastfeeding is subject to the nature of a women’s job and occupation, especially at places where women are engaged in industrial work away from home, and long working hours [7, 8]. Elsewhere, Magner, and Phillipi attribute cessation of breastfeeding within the first month to returning to work [9]. Aryeetey and Goh note that exclusive breastfeeding in Ghana usually lasts for a median of about three months, which, incidentally coincides with the maternity leave period [10]. Cai et al. in their 2012 “global trends in exclusive breastfeeding” indicate that the early cessation of exclusive breastfeeding favours the use of commercial breast milk substitutes, often of poor nutritional quality [11]. Recently, Fosu-brefo and Arthur in their work titled “effect of timely initiation of breastfeeding on child health in Ghana” acknowledged that interventions that improved child health and prevented childhood diseases included early breastfeeding initiation [12]. Also, the factors acknowledged locally in Ghana, Ayton and colleagues have identified several others that are harmfully associated with effective breastfeeding, such as delays in and/or failure of early breastfeeding initiation [13]. Exploring the constraints to exclusive breastfeeding practice among breastfeeding mothers in Southwest Nigeria, Agunbiade and Ogunleye note that early introduction of complementary feeding, based on false beliefs that it is only beneficial to infants less than six months, adversely affects breastfeeding initiation and sustainability [14]. In China and Western Kenya, several factors accounted for low EBF prevalence among working mothers. Early return to work, limited flexibility of work hours, lack of privacy [15], as well as a feeling of being watched and judged, lack of support including networks, tiredness and emotional support at work [16] were cited as challenges facing working mothers. Mother’s work outside the home, father’s type of occupation (demanding occupations) which may limit their support for mothers to breastfeed and shorter maternity leave regulation also hindered EBF practice among professional working mothers in Vietnam [17], who all intended to exclusively breastfeed. These studies report that although most working mothers leave the maternity ward breastfeeding exclusively, the practice is quickly abandoned, mostly due to work and employment related factors. Although breastfeeding may not be completely abandoned, its exclusivity was mostly interrupted by these factors. Some of the factors hindering exclusive breastfeeding initiation and practice in Ghana and elsewhere include poor knowledge of mothers, lack of mother’s confidence, lack of skills about appropriate breastfeeding methods and challenges with other work problems during lactation [16, 18, 19]. These challenges may be amplified among working mothers in Ghana, and could include giving substitutes other than maternal milk, early introduction of weaning foods, or shorter duration of EBF due to demands from work.

While data indicate that only about 36 % of infants younger than six months are exclusively breastfed in developing countries [20], national surveys concluded that Ghana’s exclusive breastfeeding rate at six months is currently about 52 % [18]. Although higher than the national average, the exclusive breastfeeding prevalence of 60 % in the Upper West Region is lower than the desired national target. The popularity or otherwise of exclusive breastfeeding among gainfully employed women is yet to be characterized in this region of Ghana. The current study therefore aimed to assess the prevalence, and predictors of exclusive breastfeeding among professional working mothers in the Upper West Regional capital of Ghana.

## Methods

### Study design, area, population, and inclusion criteria

The study was a descriptive cross-sectional survey and employed only quantitative data collection methods. The study was done in Wa, the capital of the Upper West Region, in the north west of Ghana. The Wa Municipal is one of the nine districts in the Upper West Region and its capital is also the regional capital, which attained its municipal status in August 2004. The population of the region in 2014, based on the year 2010 Population and Housing Census with a projected growth rate of 1.9 % is 702,110 [5]. This study involved professional working mothers resident in the capital, Wa. This study defined professional working mothers as women who are employed in the formal sector. They were eligible to partake in the study if they had infants/children between the ages of six (6) and 24 months. As indicated within this paper, the researcher’s choice of professional working mothers for the study was determined by the limited data on exclusive breastfeeding among this group of women [21].

### Sampling technique and sample size

A systematic random sampling technique was used with the register of females employed in the formal sector (18,021) in the Municipality as the sampling frame. This sampling frame was gained from all institutions under the formal sector, comprising both public and private sectors. Formal sector as defined by this study, encompasses all jobs with usual hours (8 am to 5 pm) attracting regular wages, and are recognized as income sources, on which income taxes is paid.

The sample size for the study was calculated based on the prevalence of EBF in the general population and others. The sample size for this study was calculated as follows:

*n* = Z2 × PQ/d2, where n represents the desired sample size, Z is the normal standard deviate, whose value at 95.0 % confidence level is 1.96, P = current EBF rate; 0.6 [16, 22], Q = 1-P = 0.4, and d = the set margin of error; 0.05. Thus minimum sample size, *n* = 368.7936 or *n* = 369. The figure was upwardly adjusted by 5 % to cater for possible non-respondents or recording errors. The resultant sample size was 387.

The total population (18,021) was divided by the estimated sample size (387). A number between 1 and 46 was randomly generated using a random integer generator at www.random.org [23] to serve as the random start point. After a random start, every 46th person was picked, her corresponding institution identified and she was interviewed. This was repeated until the sample size of 387 was reached. It is worthy of note that the upwardly adjusted sample size of 387 could not be met. The single most important determinant of our inability to interview the 387 was time. As a time-bound graduate work, when the data collection period was exceeded by some weeks without the desired numbered, a decision was taken to conclude the data collection when the minimum sample size of 369 was met. The 5 % adjustment to address potential non-response and recording errors was deemed inconsequential.

### Data collection

The researchers interacted with the mothers using a structured interviewer administered questionnaire. The consent of the mothers was sought prior to the commencement of the questionnaire administration. Once mothers were identified to be eligible, they were invited orally, to participate in the interview. Of 375 mothers deemed eligible to participate in the study, 369 consented. This gives a 98 % response rate.

#### Data collection tools and procedures

Before the start of the data collection, research assistants were recruited and orientated. They were guided on the purpose of the research, the focus of the research, the administration of completing questionnaires without any form of coercion, and handling of unresponsive interviewees during the process. The structured questionnaires were then distributed to the research assistants for administration over a period of one month. The questionnaires consisted of five sections. The first part included questions on participants’ socio-demographic characteristics and breastfeeding; the second section assessed mothers’ awareness of exclusive breastfeeding. The third and fourth sections included the exclusive breastfeeding practices of mothers and employment related factors influencing exclusive breastfeeding respectively. The last section sampled the views of mothers regarding the health workers recommendations pertaining to exclusive breastfeeding. All variables presented were coded with numeric values.

### Data analysis

The data entry and analysis were performed using the International Business Machines Statistical Package and Service Solutions, IBM SPSS version 20 data processing software. Administered questionnaires were collated at the end of each day. All necessary differences and errors were rectified before the processing. The data were then processed afterwards into tables to show the frequency and percentages of the distribution of the data. Uni-variate analysis was used to generate frequencies. The main outcome, the exclusive breastfeeding rate, was measured using three standard questions. First, mothers answered a question on what age of the child they started giving other foods and drinks to their infants, secondly, they were asked how many months they breastfed baby with only breast milk, and thirdly, whether they (mothers) have ever given anything to the child to eat or drink apart from breast milk before the child was six months old.

Simple logistic regression analysis was done to further test the strength and direction of the association between several independent variables and the outcome variable. In a multiple logistic regression model, adjusted odds ratios (AOR) were calculated to control for confounders. Variables with *p* < 0.25, at the simple logistic regression analysis were selected into the multiple regression models as per [24, 25]. Several factors were considered potential factor including the type of delivery mothers had, feeding method most difficult to practice, infant feeding recommendation by health workers at birth and duration of maternity leave. Adjusted odds ratios were used to control confounders in a multiple logistic regression model. Confounders assessed included but not limited to educational background, type of delivery, duration of maternity leave, and advice from mothers’ support persons. *P* < 0.05 denotes statistical significance.

### Ethical approval and consent to participate

Ethical clearance was obtained from the Ethics Committee of the Ghana Health Service (Ethical Approval ID # for the study is GHS-ERC:91/02/15). Permission was obtained from the Wa Municipal Health Director of Health Services, and from the various heads and representatives of the working establishments in Wa Central. Informed consent was obtained from all participants after the objectives and the methodology of the study was explained to them. Participation in the study was completely voluntary, no financial or material benefits were given. The privacy and confidentiality of every participant was ensured throughout the study period.

## Results

### Descriptive results

There were 369 study participants, consisting of professional working mothers who had children aged between 6 and 24 months. Most (48 %) of the respondents were between the ages 24 and 30 years; about 10 % were 36 years or older. Overall, one in every three of the children was aged between 18 months and 24 months; 18 % between 9 months and 11 months. More than 90 % of the participants had attained tertiary education. The Northern ethnic groups (Dagaari, Waali, Sissali, Dagbani, Frafra, Kassin and Mamprusi) formed the majority (85 %) of the respondents as compared to Southern ethnic groups (Ezema, Ewe, and Akan). Other details are indicated in Table 1.

**Table 1:** Background attributes of respondents (*n* = 369)

Attribute	Frequency	Percent
Age of respondent (in years)		
24–30	177	48.0
31–35	158	42.8
36 or older	34	9.2
Age of child (in months)		
6–8	72	19.5
9–11	68	18.4
12–17	96	26.0
18–24	133	36.0
Marital Status		
Married	343	93
Not married	26	7
Highest level of schooling completed		
Voc/Technical	26	7
Tertiary	343	93
Mother tongue of respondents		
Southern Languages	56	15.2
Northern Languages	313	84.8
Religious denomination of respondents		
Christian	235	63.7
Muslim	134	36.3
Place of work of respondents		
Outside home	359	97.3
Home	10	2.7
Type of employment		
Full time	360	97.6
Part time	9	2.4
Gestation at birth		
Premature (less than 9 months)	11	3
Full-term (9 months)	358	97
Type of delivery		
Normal delivery	352	95.4
Caesarean/section	17	4.6

### Mothers’ awareness of exclusive breastfeeding and feeding practices

The study reveals a near universal awareness on exclusive breastfeeding; 99 % of them knew that a child should be breastfed for the first six months. Almost all mothers (94 %) were aware that infants are healthier on only breast milk for the first six months than they do on other milks. Although awareness about EBF was very high, its practice was low. Only 10.3 % had exclusively breastfed their infants for six months. Of note however, is the fact that most (91 %) of the respondents initiated breastfeeding within the first hour after delivery. A little above half (52 %) of the respondents gave water to their infants before they were six months old. Other details are supplied in Table 2.

**Table 2:** Mothers’ awareness of exclusive breastfeeding

Attributes	Frequency	Percent
Awareness that babies need to be breastfed for the first six months	366	99.2
Believes that infants do better on only breast milk for the first six months.	346	93.8
Sources of education on exclusive breastfeeding		
Nurses/health worker	275	74.5
Media/Other sources	94	25.5
Initiation of breastfeeding		
Within the first one hour	336	91.1
After one hour	31	8.4
Ever breastfed baby	364	98.6
Introduction of child to other foods/milks		
Less than six months	311	89.7
From six months	38	10.3
Foods given to infants before six months		
Water	191	51.8
Porridge	106	28.7
Lactogen	34	9.2
Milk products usually used for feeding		
Tin milk (lactogen infant formula)	95	25.7
Breast milk	213	57.7
Cerelac infant formula	17	4.6
SMA	8	2.2
None	22	6.0
Others (Ideal milk, Carnation, cowbell)	14	3.8
Feeding method most convenient for mothers		
Breastfeeding	353	95.7
Formula feeding	16	4.3
Feeding method most difficult for mothers to practice		
Breastfeeding	55	14.9
Formula feeding	314	85.1
Health workers recommendation on infant feeding from birth		
Breast milk	346	93.8
Formula foods	23	6.2
No recommendation	5	1.4
Nurses’ recommendations on infant feeding beyond six months.		
Breast milk	15	4.1
Complementary feeding (semi-solid, porridge, family foods)	340	92.1
Formula feeding	3	.8
No recommendation	11	3.0
Mothers had maternity leave with pay	356	96.5
Support from worksite to continue breastfeeding after maternity leave		
Not at all	263	71.3
Some support	106	28.7
Mothers allowed to have their children at the work place	176	47.7
Mothers who had breastfeeding rooms at their work place	16	4.3
Mothers given special break for breastfeeding	81	22.0
Mothers on shift duties	38	10.3

### Exclusive breastfeeding and health workers’ recommendations and mothers’ work environment

Almost all (98 %) of the responses indicated that health workers recommended breast milk to mothers as the best food for children for the first six months. The study also explored the mothers’ work environment in relation to exclusive breastfeeding. Most (81 %) of the mothers had three months maternity leave. Beyond this, many mothers (69 %) did not receive any support from their employers to help them continue breastfeeding (Table 2).

### Factors associated with exclusive breastfeeding (unadjusted associations)

Odds ratios (OR) were calculated to determine the strength of association between independent variables and EBF (Table 2). The results indicate that, age of the respondents, marital status, level of education, and respondents’ language were not associated with mothers’ practice of exclusive breastfeeding. Mothers who were pregnant for less than nine months (OR 0.11; 95 % CI 0.02, 0.80) as compared to mothers who had a full time pregnancy were less likely to practice exclusive breastfeeding. Mothers who went through a normal delivery compared to caesarian delivery, were almost ten times as likely to practice exclusive breastfeeding (OR 9.54; 95 % CI 3.43, 26.54). The odds of practicing EBF were high among mothers who were aware that infants need to be exclusively breastfed for the first six months (OR 14.10; CI 2.28, 87.27). The mothers who were advised by their support persons to include formula feeding were less likely to engage in the practice of exclusive breastfeeding (OR 0.19; CI 0.05, 0.79). Mothers who considered formula feeding as the most difficult to practice were more likely to adopt exclusive breastfeeding (OR 2.27; CI 1.03, 4.99).

Recommendations given at birth by health workers were also assessed. The odds of practicing exclusive breastfeeding among mothers who were advised to use formula feeding was low (OR 0.18; CI 0.07, 0.45). Three months or longer durations of maternity leave was also significantly associated with a mother’s practice of exclusive breastfeeding. Respondents who were on maternity leave for less than three months were less likely to have exclusively breastfed (OR 0.14; CI 0.03, 0.66). Of note, however, is the fact that all other variables did not have any association with mothers’ practice of EBF.

### Factors associated with exclusive breastfeeding (multivariate analyses)

In a multiple logistic regression model, adjusted odds ratios (AOR) were calculated to control for confounders. Variables with *p* < 0.25, at the simple logistic regression analysis were selected into the multiple regression models. After controlling for a number of covariates, normal delivery (AOR 9.02; CI 2.85, 28.53), the type of infant feeding recommendation by health workers at birth (AOR 0.01, CI 0.27, 0.77), three months or longer duration of maternity leave (AOR 0.09; CI 0.02, 0.45), and feeding method most convenient to mothers (AOR 3.13; CI 0.96, 10.23) were all independently associated with exclusive breastfeeding (Table 3).

**Table 3:**
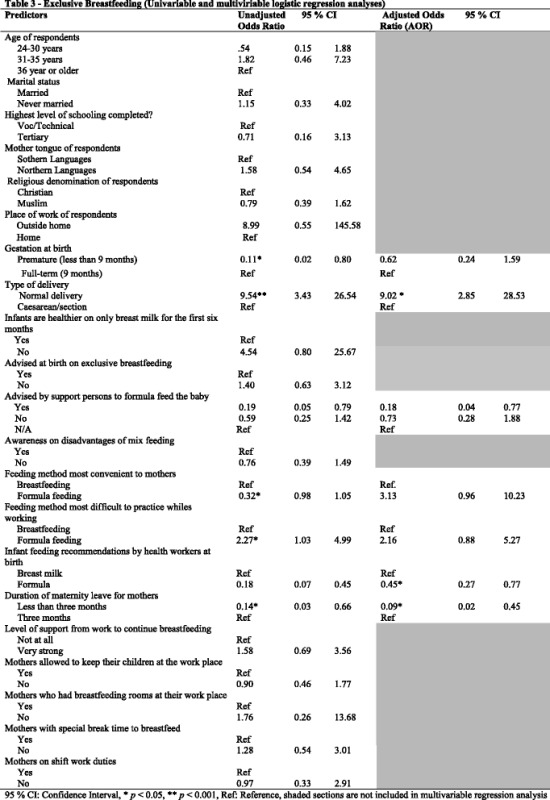
Exclusive Breastfeeding (Univariable and multiviriable logistic regression analyses)

95 % CI: Confidence Interval, * *p* < 0.05, ** *p* < 0.001, Ref: Reference, shaded sections are not included in multivariable regression analysis

## Discussion

Data from this study show that although awareness on exclusive breastfeeding among professional working mothers is almost universal (99 %), the practice of EBF at six months is low (10.3 %). Elsewhere, Al-binali [26] found 89 % of mothers had a good knowledge about exclusive breastfeeding but only a small percentage (8.3 %) engaged in the practice for the first six months. The work of Afrose et al. in Dhaka City among working mothers reported findings contrary to those of this study [27]. Afrose et al. showed that most working mothers who received their main source of information from nurses and other health workers (75 %) were not EBF. Data from work done by Anyanwu and Maduforo in 2004 [28] in a neighboring country, and others [29] concur that the main source of education about breastfeeding provided to working mothers is the health worker, although determinants of EBF among working mothers include the women’s educational status and working conditions [30, 31]. Februhartanty and colleagues presented data similar to those of the current study [30]. They found that all working mothers knew about exclusive breastfeeding and the need to breastfeed infants for the first six months. Others have suggested that the enhancement of education and knowledge among working mothers can improve workplace lactation [32]

The importance of early initiation of breastfeedingis well recognised [2, 30, 33]. In the current study, nearly all working mothers (91 %) initiated breastfeeding within the first one hour of delivery. This concurs with the results of Heck's study where a high rate 95.2 % of working mothers had initiated breastfeeding within one hour after birth [34], but Liben & Yesuf, in Ethiopia observed only 39.6 % breastfeeding initiation rate among working mothers [35]. In Ghana, 56 % started breastfeeding within the first one hour of birth [30]. This figure is rather low as compared to the finding of this study (91 %) and that of the general population in the Upper West Region which recorded 60 % [16]. This disparity in the prevalence of early breastfeeding initiation could be attributed to the potentially high level of exposure of EBF among the current group of relatively highly educated women, in comparison to their counterparts in the general population.

Even though breastfeeding initiation is high, more than 50 % of women of child bearing age are employed, and most return to work at a time when exclusive breastfeeding is ideal [36]. This results in reduced practice of EBF among working mothers within the first six months. Possible hindrances to the sustained practice of exclusive breastfeeding among professional working mothers as indicated by this study include conflict of commitment, limited workplace support, lack of breastfeeding facilities and short or lack of official breaks. Thus, the demand to perform official duties is difficult with the obligation to breastfeed at the same time. This finding is similar to other studies where infants of working mothers failed to attain their full breastfeeding and health potential because they were denied the option to breastfeed while at work [37]. Considering these challenges, several studies have cited paid extended maternity leave, appropriate institutional policies and lactation breaks as proven to be the most effective interventions in promoting breastfeeding among working mothers [36, 38, 39]. In other studies done in Ghana, maternity leave was cited as having a positive breastfeeding duration among working mothers [8]. It is worthy of note that, in Ghana, this period is limited to only three months by law, which is far too short for a working mother to practice exclusive breastfeeding to six months, making the practice a challenge. In Western Pacific settings, maternity leave extends to sixteen weeks [2] but other studies in Taiwan cited that most companies provide only eight weeks of maternity leave [40].

With limited flexibility and workplace support indicated by this study, formula supplementation seems unavoidable for many working mothers, even though their preference may still be to exclusively breastfeed. Similarly, included among experiences in Ireland, working mothers experienced negative attention or a lack of support due to their continued breastfeeding while being back to work [41]. The practice most convenient for mothers in this study was breastfeeding. Contrary to this recommended practice, professional working mothers elsewhere failed to adhere to breastfeeding recommendations, with the impression that the practice is not welcomed within workplaces [42]. Those who said breastfeeding practice was the most difficult to combine with work were twice as likely to practice formula feeding (OR 2.27; 95 % CI 1.03, 4.99). In the same perspective, other evidence suggests that breastfeeding is more likely compromised among professional working [6].

An earlier study by Adeyinka et al. showed that maternity ward practices and the health professionals' advice were associated with exclusive breastfeeding [7]. However, advice given by nurses on how to practice EBF did not influence exclusive breastfeeding (OR 1.40; 95 % CI 0.63, 3.12) in the current study. Support and counseling from health care providers can tremendously improve breastfeeding practices [43].

The most important factor that determines EBF practice among professional working mothers is maternity leave (OR 0.14; 95 % CI 0.03, 0.66). Using a multivariable regression analysis, the duration of maternity leave was an independent predictor of exclusive breastfeeding practice (OR 0.09; 95 % CI 0.02, 0.45). It is worth noting that earlier findings by Ali and Ayed confirm this finding, where mothers’ successful practice of EBF was attributed to a three months maternity leave [43]. Further studies shared similar findings where mothers are more likely to breastfeed only during the three months maternity leave [40].

This study is not without limitations. One limitation of this study is the inclusion of only professional working mothers. This could have resulted in the high level of awareness recorded by the study, since all respondents in the study had attained some level of education. The study was also limited to only Wa Municipality, which was a small part of the region. This limits the generalizability of the findings. Another limitation of the study is recall bias. Since mothers had already experienced the exclusive breastfeeding period, it would have been difficult for mothers to remember all experiences during the period. The study is also likely to be under powered.

## Conclusions

Among this group of professional working mothers, breastfeeding initiation at birth was near universal, although sustained and exclusive breastfeeding till the sixth month of life was low at 10.3 %. Mothers were well informed about exclusive breastfeeding and its benefits; however, this knowledge did not translate into practice. Based on the findings, professional working mothers may require more supportive post-delivery follow ups. Also, policies aimed at improving EBF rates among professional working mothers should include maternity leave extension and educational programs that encourage knowledge into practice. Collaboration between various stakeholders including Ghana Health Service, Ministry of Health, Department of Employment and Labour Relations, and all heads of professional organizations/institutions may yield the breastfeeding-friendly policies and breastfeeding-sensitive work environments being advocated for by this work.

## Abbreviations

AOR: Adjusted odds ratio
EBF: Exclusive breastfeeding
OR: Odds ratio
CI: Confidence interval
ERC: Ethics Review Committee
GDHS: Ghana Demographic and Health Survey
GHS: Ghana Health Service
IBM: International Business Machines
SPSS: Statistical Package for Service Solutions
